# Supplementary material for: Isothiocyanate-Rich Extracts from Cauliflower (Brassica oleracea Var. Botrytis) and Radish (Raphanus sativus) Inhibited Metabolic Activity and Induced ROS in Selected Human HCT116 and HT-29 Colorectal Cancer Cells
Source: Int J Environ Res Public Health. 2022 Nov 13;19(22):14919. doi: 10.3390/ijerph192214919 (PMC9691161; doi:10.3390/ijerph192214919)
Supplement: Supplementary file 1 [file ijerph-19-14919-s001.zip › ijerph-1954245-supplementary.pdf]

International Journal of
Environmental Research and Public Health

MDPI

Supplementary File

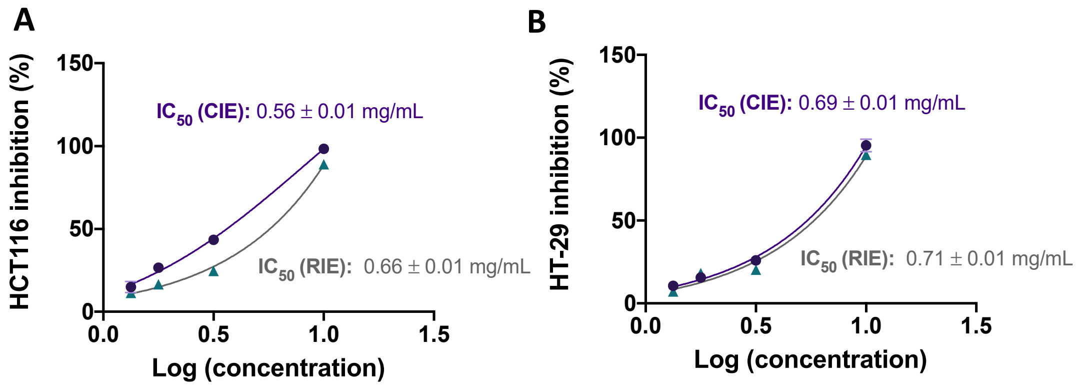

Supplementary Figure S1. Three parameters dose-response curves of CIE and RIE treatments for: **(A)** HCT116 and **(B)** HT29 cells.

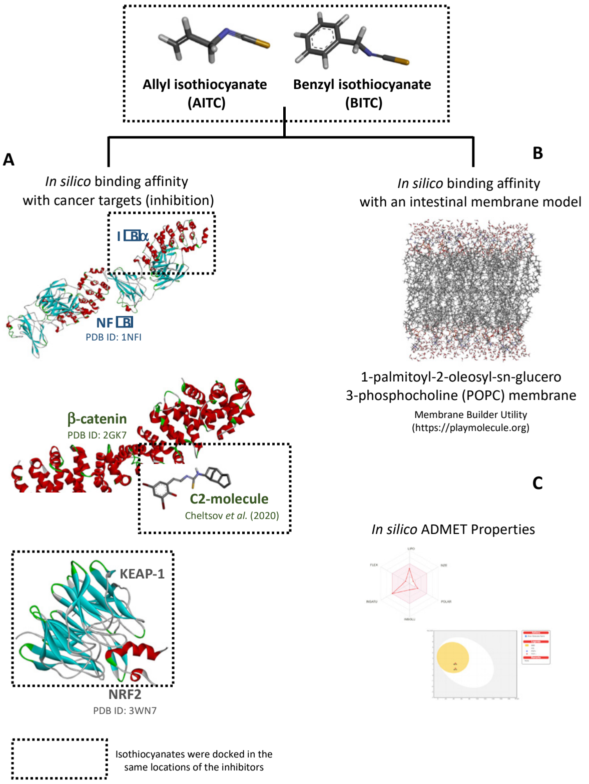

Supplementary Figure S2. Overall scheme of the *in silico* docking procedure.

Isothiocyanates (Allyl isothiocyanate of AITC; benzyl isothiocyanate of BITC) were downloaded from PubChem Database (AITC PubChem CID: 5971; BITC PubChem CID: 2346) and used as ligands, that were prepared in Discovery Studio Visualizer. Then, two *in silico* approaches were used: (A) ligands were docked in the same position of inhibitors from the cancer target molecules: IκBα, C2-molecule, and KEAP-1, respectively for NFκB, β-catenin, and NRF2; (B) Ligands were docked in a simulation of intestinal permeation using a generated 1-palmitoyl-2-oleosyl-sn-glycero-3-phosphocholine (POPC) membrane, that was generated in the Membrane Builder utility from https://playmolecule.org (accessed on 18 September 2022); (C) The verification of the *in silico* ADMET properties of

the ligands was screened through bioinformatic utilities (admetSAR 2.0 and SwissADME online software).

Supplementary Table S1. Validation of HPLC method using for the identification and quantification of isothiocyanates.

Standard Name	Detection Wavelength (nm)	RT (min)	Linearity Range (μg/mL)	Regression Coefficient (R^2)	LOD (μg/mL)	LOQ (μg/mL)
AITC	240	21.51	0-100	0.991	2.05	2.24
BITC	240	37.85	0-100	0.996	2.10	2.38

AITC: Allyl isothiocyanate; BITC: benzyl isothiocyanate; RT: retention time; LOD: Limit of detection; LOQ: limit of quantification.

Supplementary Table S2. Results from the recovery test of isothiocyanates.

Standard Name	Amount Tested	Initial Amount (μg/ml)	Found Amount (μg/mL)	Recovery (%)	Average Recovery (%)	RSD (%)
AITC	Low	15.20	14.89	97.96	98.69	0.79
	Medium	35.71	35.21	98.59		
	High	60.22	59.93	99.52		
BITC	Low	14.90	14.88	99.87	99.27	0.64
	Medium	40.23	39.67	98.61		
	High	62.14	61.72	99.32		

AITC: Allyl isothiocyanate; BITC: benzyl isothiocyanate; RSD: Relative standard deviation.
